# SimReadUntil for benchmarking selective sequencing algorithms on ONT devices

**DOI:** 10.1093/bioinformatics/btae199

**Published:** 2024-04-11

**Authors:** Maximilian Mordig, Gunnar Rätsch, André Kahles

**Affiliations:** Biomedical Informatics Group, Department of Computer Science, ETH Zurich, Zürich, 8092, Switzerland; Empirical Inference, Max Planck Institute for Intelligent Systems, Tübingen, 72076, Germany; Biomedical Informatics Group, Department of Computer Science, ETH Zurich, Zürich, 8092, Switzerland; Biomedical Informatics Research, University Hospital Zurich, Zürich, 8091, Switzerland; Swiss Institute of Bioinformatics, Lausanne, 1015, Switzerland; Department of Biology, ETH Zurich, Zürich, 8092, Switzerland; Biomedical Informatics Group, Department of Computer Science, ETH Zurich, Zürich, 8092, Switzerland; Biomedical Informatics Research, University Hospital Zurich, Zürich, 8091, Switzerland; Swiss Institute of Bioinformatics, Lausanne, 1015, Switzerland

## Abstract

**Motivation:**

The Oxford Nanopore Technologies (ONT) ReadUntil API enables selective sequencing, which aims to selectively favor interesting over uninteresting reads, e.g. to deplete or enrich certain genomic regions. The performance gain depends on the selective sequencing decision-making algorithm (SSDA) which decides whether to reject a read, stop receiving a read, or wait for more data. Since real runs are time-consuming and costly, simulating the ONT sequencer with support for the ReadUntil API is highly beneficial for comparing and optimizing new SSDAs. Existing software like MinKNOW and UNCALLED only return raw signal data, are memory-intensive, require huge and often unavailable multi-fast5 files (≥100GB) and are not clearly documented.

**Results:**

We present the ONT device simulator SimReadUntil that takes a set of full reads as input, distributes them to channels and plays them back in real time including mux scans, channel gaps and blockages, and allows to reject reads as well as stop receiving data from them. Our modified ReadUntil API provides the basecalled reads rather than the raw signal, reducing computational load and focusing on the SSDA rather than on basecalling. Tuning the parameters of tools like ReadFish and ReadBouncer becomes easier because a GPU for basecalling is no longer required. We offer various methods to extract simulation parameters from a sequencing summary file and adapt ReadFish to replicate one of their enrichment experiments. SimReadUntil’s gRPC interface allows standardized interaction with a wide range of programming languages.

**Availability and implementation:**

Code and fully worked examples are available on GitHub (https://github.com/ratschlab/sim_read_until).

## 1 Introduction

Nanopore sequencing has emerged as a promising third-generation sequencing technology that delivers long reads at an affordable price. An Oxford Nanopore Technologies (ONT) sequencer consists of a flowcell, which has a set of channels that sequence reads in parallel. As a read goes through a biological pore, it produces a characteristic current pattern that can be converted to its corresponding nucleotide sequence. By applying a reverse voltage to the pore, a read can be (r)ejected. After 72 h of sequencing, the flowcell usually needs to be replaced (costing around 500$). Unlike other sequencing technologies, ONT devices support the ReadUntil API (Loose *et al.* 2016) which gives real-time access to the read in-progress and allows to perform actions on the sequencer: reject a read, stop receiving data from a read but keep sequencing it (to reduce processing overhead), or no action to wait for more data from a read (by default). *Selective sequencing*/*adaptive sampling* leverages the ReadUntil API to enrich target sequences or deplete non-target sequences by delegating the decisions on which actions to perform to a selective sequencing decision-making algorithm (SSDA). An SSDA typically has various tunable parameters, e.g. the trade-off between a high-accuracy (compute-intensive) and a low-accuracy (fast) basecaller, the number of basepairs to take into account for read mapping, the number of mapping attempts, or the hyperparameters of a learning algorithm. Simulating the ONT device can help with tuning the SSDA parameters by reducing sequencing cost and accelerating the parameter optimization through parallel execution.

Existing simulators focus on generating reads at the raw signal level (Li *et al.* 2018) or nucleotide level (Yang *et al.* 2017). They do not respond to selective sequencing decisions and do not model the read gaps or reduced pore throughput due to pore blockage. The only available ONT simulators with ReadUntil support are ONT’s MinKNOW (https://nanoporetech.com/about-us) for-research-only playback function, UNCALLED (Kovaka *et al.* 2021), and Icarust (Munro *et al.* 2023). The former two do not permit to effectively evaluate SSDAs because they are memory-hungry and require raw signals which are both huge and often unavailable. Table 1 and Supplementary Fig. S1 give a detailed comparison of existing simulators. The concurrent work Icarust (Munro *et al.* 2023) is an implementation in Rust related to ours, but returns raw signal level data rather than basecalled data. It does not extract parameters from an existing run, assumes constant gaps between reads, does not offer acceleration and mux scans, and simulates perfect reads only. It is still in preliminary state and not suited for evaluation purposes since it cannot be started/stopped on demand and does not accept existing raw signal data as input. To predict enrichment levels prior to running a sequencing experiment, Martin *et al.* (2022) present a simple mathematical model to estimate the maximum achievable relative enrichment (explained in Supplementary Section SD), not taking pore degradation into account.

**Table 1.** Condensed version of Supplementary Table S2.

	MinKNOW	UNCALLED	Icarust	*SimReadUntil*
Input: ACTG alphabet	✗	✗	**✓**	**✓**
Input: Accepts reads	✗	✗	✗	**✓**
Output: raw/basecalled reads	**✓**/**✓**	✗/✗	**✓** ^‡^/✗	✗/**✓**
Simulate arbitrary genome and extract parameters from a run of a different genome without exact replication	✗	✗	✗	**✓**
ReadUntil via gRPC	✗	✗	**✓** ^‡^	**✓** ^‡^
Acceleration/parallelizable	✗/✗	(**✓**)^†^/**✓**	✗/**✓**	**✓**/**✓**
Open source	✗	**✓**	**✓**	**✓**
Fast generation of sequencing summary (no basecalling)	✗	✗	✗	**✓**

Comparison of simulators with ReadUntil functionality. Icarust was not yet officially published, so it may still change.†: UNCALLED’s acceleration mode distorts the length of long gaps. Icarust should be used instead of the UNCALLED simulator (personal communication). ‡: The basecalled signal is converted to a raw signal using a pore model (or additionally with the memory-intensive Scrappie for Icarust).

Given the limitations of the existing simulators, the performance of SSDAs has been assessed by either evaluating the length of rejected reads and mapping speed as a proxy for selective sequencing performance (Kovaka *et al.* 2021, Sneddon *et al.* 2022, Ulrich *et al.* 2022, Firtina *et al.* 2023), or by running very few real experiments only (Kovaka *et al.* 2021; Payne *et al.* 2021). This is time-consuming, costs a non-negligible amount of money, and prevents parameter optimization. Our simulator addresses this gap and allows to evaluate and optimize existing SSDAs more thoroughly whilst being lightweight. It can extract parameters from an existing run to be close yet not too similar to avoid overfitting the parameters to a particular run.

## 2 Methods

We present SimReadUntil which takes as input a set of full reads that can come from an existing run or from a full read simulator like NanoSim (Yang *et al.* 2017), see Supplementary Fig. S2. The reads may include barcode sequences. The (shuffled) full reads are distributed to the channels and short and long gaps are inserted between reads, where a short gap corresponds to the time for a new read to move into the pore and a long gap signifies a temporarily inactive channel. A channel can also become permanently inactive, not delivering any new reads. Each read has a start delay to account for adapters which are not part of the actual read. Mux scans can be triggered which terminate the current element in-progress across all channels (and are useful for simulating ultra-long read breaking). If a long gap is active when the mux scan starts, the long gap continues after the mux scan finishes. The simulator supports the ReadUntil API (Loose *et al.* 2016). The function get_basecalled_read_chunks(channel) returns any new data from reads *in-progress* since the last time it was called, with a configurable minimum chunk size. Different from the ONT ReadUntil API, the chunks are returned in basecalled format, after an added pause to mimic the basecalling delay (proportional to the number of nucleotides). We also offer a prototype conversion to raw signal using ONT’s pore models to work with raw signal-based SSDAs (https://github.com/nanoporetech/kmer_models). A read no longer returns its chunks after the *stop_receiving* action has been called on it, yet the full read is still written to disk. The *unblock* (reject) action terminates a read and adds an extra unblock delay right after it. If the read is no longer present by the time the action is processed, the action is logged as missed and signaled through the return value of the action call. Whenever a batch of reads has finished, the reads are written as FASTA records to disk. The FASTA header includes information such as the original full read id, the channel, and the reason the read was ended, which is useful for post-processing and for later read mapping. The FASTA header contains the metadata to construct a sequencing summary file with the essential columns. For rejected reads with a NanoSim read id, the id is modified to reflect the effective reference length. The simulation can be accelerated and issues a warning if it cannot keep up with the desired acceleration. The ReadUntil API is supplemented with a gRPC implementation which makes the simulator usable from a wide range of programming languages and allows decoupling the simulator from the SSDA through interprocess communication.

Each channel collects statistics such as the number of reads, basepairs, rejections, missed rejections. They are output at regular intervals. Reads may interact with the pore which can deteriorate the pore’s health over time and results in longer read gaps, so it is important to model non-constant read gaps. The gaps are sampled from a GapSampler that receives the channel statistics as input. We provide several gap samplers that determine their parameters from an existing run. One gap sampler imitates an existing run (similarly to UNCALLED), another produces constant or random gaps for prototyping, yet another aggregates gaps from all channels and then samples them in fixed windows. Our recommended gap sampler rolling_window_per_channel samples the channel first (from the original run) and then samples gaps from a rolling window. New gap samplers can be easily added. See the Supplemental Material for more details.

The simulator enables benchmarking and hyperparameter tuning of selective sequencing algorithms. The hyperparameters can be tuned to different ONT devices, e.g. a GridION with a GPU can compute more than a portable MinION/Flongle that relies on an external computer. Different policies for changing hyperparameters over the course of a run can also be tested. Moreover, new actions, such as stopping to receive data for a limited amount of time only, can be tested as a proof of concept for what real ONT devices should support.

## 3 Results

As opposed to MinKNOW and Icarust, our simulator is light-weight, so we run it on 2 cores of an Intel(R) Xeon(R) Gold 5220 CPU 2.20 GHz with 8GB of RAM and 50GB of SSD disk. We ran one experiment comparing the different gap samplers and another that shows how to combine our simulator with ReadFish. We find that the constant_gaps method is easy to implement, but not very realistic except for assessing SSDAs handling peak throughput (Supplementary Figs S5–S7). The gap_replication is the best method, but almost exactly reproduces a run, so time-dependent (blackbox machine learning based) SSDAs may memorize this structure and perform worse on a new run. Therefore, the recommended rolling_window_per_channel method represents a good trade-off between giving similar throughput and showing varying behavior at a smaller timescale. Similar to (Payne *et al.* 2021), we reproduce the enrichment of human chromosomes with ReadFish connected to our simulator, as shown in Supplementary Figs S8 and S9. We compute the relative and absolute enrichment predicted by the simulator and comment on reasons for discrepancies with respect to ReadFish’s reported results and the maximum achievable relative enrichment computed by the model in (Martin *et al.* 2022). Despite a sim2real gap, our simulator can be used to identify good selective sequencing algorithms by looking at relative enrichment. As an example, we demonstrate that ReadFish can handle higher acceleration factors up to some point and breaks down thereafter in terms of absolute and relative enrichment. We additionally show how to replace minimap2 alignment (Li 2018) with ground-truth alignment to allow for higher acceleration factors.

## 4 Conclusion

We presented SimReadUntil to simulate an ONT device with support for the ReadUntil API, accessible both directly and via gRPC from a wide range of programming languages. The simulator only needs FASTA read files as input, which are widely available and much smaller than bulk files or multi-fast5 files. Unlike other approaches which require often unavailable bulk or raw fast5 files, its parameters can be tuned based on a (comparatively small) sequencing summary file. By avoiding the basecalling step, the simulator allows to focus on the SSDA itself and removes the need for a GPU required by modern basecallers. This is beneficial when tuning approaches like ReadFish and ReadBouncer which operate directly on basecalled data.

We plan to use the simulator to establish a benchmark repository, optimize the hyperparameters of tools like ReadFish, and compare the performance on a real run with respect to default parameters. We are confident that our simulator can reliably identify SSDAs with good real-world performance. The modular design allows future work to come up with better gap samplers as needed, e.g., to model pore deterioration caused by rejections.

## Supplementary Material

btae199_Supplementary_Data

## Data Availability

The data for the usecases presented in the supplemental material is available at https://public.bmi.inf.ethz.ch/user/mmordig/ont_project/prepackaged/usecase_data.tar.gz and was preprocessed from https://s3-us-west-2.amazonaws.com/human-pangenomics/T2T/CHM13/assemblies/analysis_set/chm13v2.0.fa.gz, https://labshare.cshl.edu/shares/schatzlab/www-data/UNCALLED/simulator_files/20190809_zymo_seqsum.txt.gz.
