# Supplementary material for: SimReadUntil for benchmarking selective sequencing algorithms on ONT devices
Source: Bioinformatics. 2024 Apr 11;40(5):btae199. doi: 10.1093/bioinformatics/btae199 (PMC11065473; doi:10.1093/bioinformatics/btae199)
Supplement: btae199_Supplementary_Data [file btae199_supplementary_data.pdf]

Bioinformatics, Year, 1–13
doi: DOI HERE
Advance Access Publication Date: Day Month Year
Paper

Supplemental Material: SimReadUntil for Benchmarking Selective Sequencing Algorithms on ONT Devices

Maximilian Mordig,[1,2]* Gunnar Rätsch[1,3,4,5] and André Kahles[1,3,4]*

[1]Biomedical Informatics Group, Department of Computer Science, ETH Zurich, Switzerland, [2]Empirical Inference, Max Planck Institute for Intelligent Systems, Germany, [3]Biomedical Informatics Research, University Hospital Zurich, Switzerland, [4]Swiss Institute of Bioinformatics, Switzerland and [5]Department of Biology, ETH Zurich, Switzerland

*Corresponding authors. {andre.kahles,maximilian.mordig}@inf.ethz.ch

FOR PUBLISHER ONLY Received on Date Month Year; revised on Date Month Year; accepted on Date Month Year

Abstract

Motivation: The Oxford Nanopore Technologies (ONT) ReadUntil API enables selective sequencing, which aims to selectively favor interesting over uninteresting reads, e.g., to deplete or enrich certain genomic regions. The performance gain depends on the selective sequencing decision-making algorithm (SSDA) which decides whether to reject a read, stop receiving a read, or wait for more data. Since real runs are time-consuming and costly, simulating the ONT sequencer with support for the ReadUntil API is highly beneficial for comparing and optimizing new SSDAs. Existing software like MinKNOW and UNCALLED only return raw signal data, are memory-intensive, require huge and often unavailable multi-fast5 files ($\geq$ 100GB) and are not clearly documented.
Results: We present the ONT device simulator *SimReadUntil* that takes a set of full reads as input, distributes them to channels and plays them back in real time including mux scans, channel gaps and blockages, and allows to reject reads as well as stop receiving data from them. Our modified ReadUntil API provides the basecalled reads rather than the raw signal, reducing computational load and focusing on the SSDA rather than on basecalling. Tuning the parameters of tools like ReadFish and ReadBouncer becomes easier because a GPU for basecalling is no longer required. We offer various methods to extract simulation parameters from a sequencing summary file and adapt ReadFish to replicate one of their enrichment experiments. *SimReadUntil*'s gRPC interface allows standardized interaction with a wide range of programming languages.
Availability: Code and fully worked examples are available on GitHub (`https://github.com/ratschlab/sim_read_until`).
Supplementary information: Supplementary data are available at Bioinformatics online.

Key words: Oxford Nanopore Technologies, Simulator, ReadUntil API, NanoSim

© The Author Year. Published by Oxford University Press. All rights reserved. For permissions, please e-mail: journals.permissions@oup.com

	MinKNOW	UNCALLED	Icarust	***SimReadUntil* (ours)**
**Inputs/outputs:**				
Input: ACTG alphabet (rather than raw signal)	✗	✗	✓	✓
Input: Accepts reads (rather than assembly)	✗	✗	✗	✓
Output: raw / basecalled reads	✓/ ✓	✗/ ✗	✓‡ / ✗	✗/ ✓
**Parameter extraction:**				
Simulated genome can be chosen	✗	✓	✓	✓
Gap extraction from an existing run without exact replication	✗	✗	✗	✓
**ReadUntil:**				
stop_receiving / *unblock* (continues with new read)	✓/ ✗	✓/ ✓	✓/ ✓	✓/ ✓
Supports ReadUntil via gRPC	✗	✗	✓‡	✓‡
**For benchmarking / efficient hyperparameter tuning:**				
Run can be accelerated	✗	(✓)†	✗	✓
Can run in parallel	✗	✓	✓	✓
**Source code:**				
Open source	✗	✓	✓	✓
Code well documented	-	✗	✓	✓
Unit / integration tests	-	✗/ ✗	✗/ ✗	✓/ ✓
Maintained	-	✗†	✓	✓
**Tools for assessment (of SSDA):**				
Fast generation of sequencing summary (no basecalling)	✗	✗	✗	✓
Generates ground-truth simulator statistics	✗	✗	✗	✓
Diagnosis plots based on ground-truth alignment	✗	✗	✗	✓⋆

Table 1. Comparison of simulators with ReadUntil functionality. Simulators without any ReadUntil functionality such as squiggle simulators are not shown. UNCALLED refers to the simulator of UNCALLED. Ground-truth simulator statistics include the number of rejected basepairs. Icarust was not yet officially published, so it may still change. †: UNCALLED's acceleration mode distorts the length of long gaps. Icarust should be used instead of the UNCALLED simulator (personal communication). ‡: The basecalled signal is converted to a raw signal using a pore model (or additionally with the memory-intensive Scrappie for Icarust). ⋆: See Figures 8, 9 for a subset of generated plots as well as the supplemental files.

We first compare existing simulators, then provide a schematic of our simulator, describe the approaches for parameter extraction from an existing sequencing summary file and compare the different parameter extraction methods. Finally, we present a use case that combines our simulator with ReadFish and NanoSim.

A. Comparison of Existing Simulators

Table 1 compares existing simulators with ReadUntil capability. ONT's MinKNOW controller software ships with a for-research simulator that takes a bulk file as input and plays it back. Read rejection is not very realistic: it simply cuts the read into two reads rather than taking a new read (Munro et al., 2023). Documentation is sparse and it requires non-trivial manual setup (Ulrich et al., 2022). The simulator from UNCALLED (Kovaka et al., 2021) is the approach closest to ours and comes with a Python wrapper around its C++ code. However, it is not well documented and not tested, does not write the finished reads to disk, often segfaults, and is tightly integrated into their code, making it difficult to change simulation behavior beyond the obvious. It requires a big raw fast5 file (comprising several tens of GBs) and a sequencing summary file from an existing UNCALLED run, which is often unavailable. UNCALLED exactly replicates the channel activity pattern of active and inactive periods for a given channel and recycles reads which effectively means that no novel data is acquired since a selective sequencing run usually has many more reads than a normal run due to rejections. Icarust was developed in the same spirit as our simulator, but has drawbacks outlined in Table 1, mostly related to computational overhead associated with raw signals and no automatic (benchmarking-like) workflow to evaluate SSDA performance. Our simulator written in Python is probably also more accessible to modification than Icarust written in Rust.

Figure 1 gives an overview of the inputs and outputs for the existing tools. With minor modification, our simulator could also take raw signals as input. Due to the high raw signal sampling rate of 4kHz per channel, this requires a significant amount of memory, disk I/O, and CPU processing (for fast5 extraction), which is relatively slow.[1] This is unnecessary when benchmarking approaches that take basecalled reads like ReadFish and ReadBouncer, where we can instead simulate the sequencing errors in advance (e.g., with NanoSim) as we demonstrate in one of the use cases.

B. Simulator Schematic

Figure 2 illustrates the design of the simulator as a collection of channels that takes full reads and outputting (full or partial) reads according to ReadUntil actions. An example of the simulator with two channels with random ReadUntil actions is given in Figure 3. The

[1] UNCALLED (Kovaka et al., 2021) mentions that their simulator can take 100GB of memory, see `https://github.com/skovaka/UNCALLED#readme`.

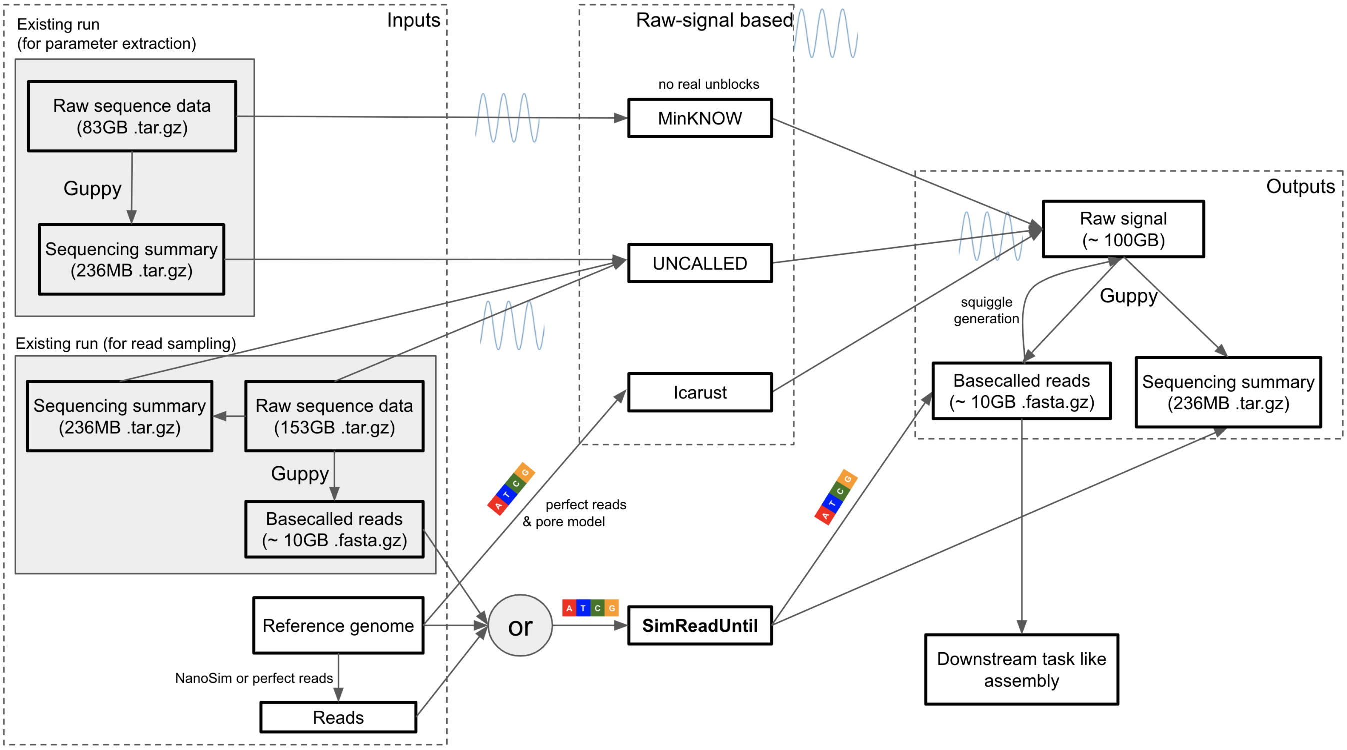

Fig. 1: Inputs and outputs for the ReadUntil simulators. *SimReadUntil* and Icarust can take a reference genome and create perfect reads from them (with no mutations and indels) which are then input to the simulator. *SimReadUntil* also takes basecalled reads as input, which can be coming from an existing run or generated from a reference genome using NanoSim. UNCALLED takes both (full) raw reads and a sequencing summary (to locate the signal in the raw files) as input. The provided file sizes are taken from the UNCALLED HMW experiment (flowcell run 1) (Kovaka et al., 2021). The original run is 153GB in size whereas the selective sequencing run to mimic is 83GB in size. It has smaller file size because the throughput during selective sequencing is reduced due to more and longer gaps. Numbers prefixed with ∼ are estimated.

simulator forwards all channels to the current time, which makes new data available via `get_basecalled_read_chunks()`. This involves (asynchronously) writing finished reads to disk and reading new (full) reads from disk. After forwarding all channels, it checks for new actions and performs them. Then, it repeats. The reads writer additionally writes a live sequencing summary file which can be used to monitor a running simulation with the provided plotting scripts. For performance reasons, we added wrappers for the reads writer (ReadsWriter) and reads reader (ReadPool) that run on separate threads and use a (threadsafe) queue-based approach to avoid blocking the main thread when reading or writing reads. This mitigates common file system latency issues.

ONT's ReadUntil gRPC implementation `get_live_reads` streams read chunks as a bidirectional gRPC stream, even when the data is not requested. We instead provide the data upon request, thereby reducing computational overhead on the simulator if data is never requested. This assumes that the gRPC connection between the simulator and the SSDA under test is fast, which is the case, especially since the basecalled data is much smaller than raw signal data. If using Python, it is possible to directly connect to the simulator without the gRPC overhead.

B.1. NanoSim & NanoSim Read IDs

We have modified the NanoSim implementation starting from commit 8341c2f665daf839b2b78982ae0f0f4d1fdec6ed to make it deterministic (in single-process mode), added the possibility to generate reads without flanking regions and made small optimizations to speed it up, e.g., the unnecessary big read error profile is not written to disk. NanoSim is a submodule of our git repository. We observed that the `median_len` and `sd_len` CLI parameters do not do what they promise. Specifying `median_len=15000, sd_len=6.9` results in a distribution with median length of 2500 bps. Therefore, we modified it to instead use a uniform read length distribution in the range `[median_len - sd_len, median_len + sd_len]` of the reference sequence. Due to indels, the resulting read can be shorter or longer. We use the pre-trained error models from NanoSim which are saved in pickle format. Due to API changes in numpy and scipy, we could not load them into the same environment as our simulator, so we decided to create a separate conda environment as suggested in their setup.

NanoSim read ids contain the ground-truth alignment information. When simulating a genome (as opposed to a metagenome) without chimeric reads, the ids are of the form:

```
[{genome}-]{chromosome}_{ref_pos}_{read_type}_{read_nb}_{strand}_{head_len}_{ref_len}_{tail_len}
```

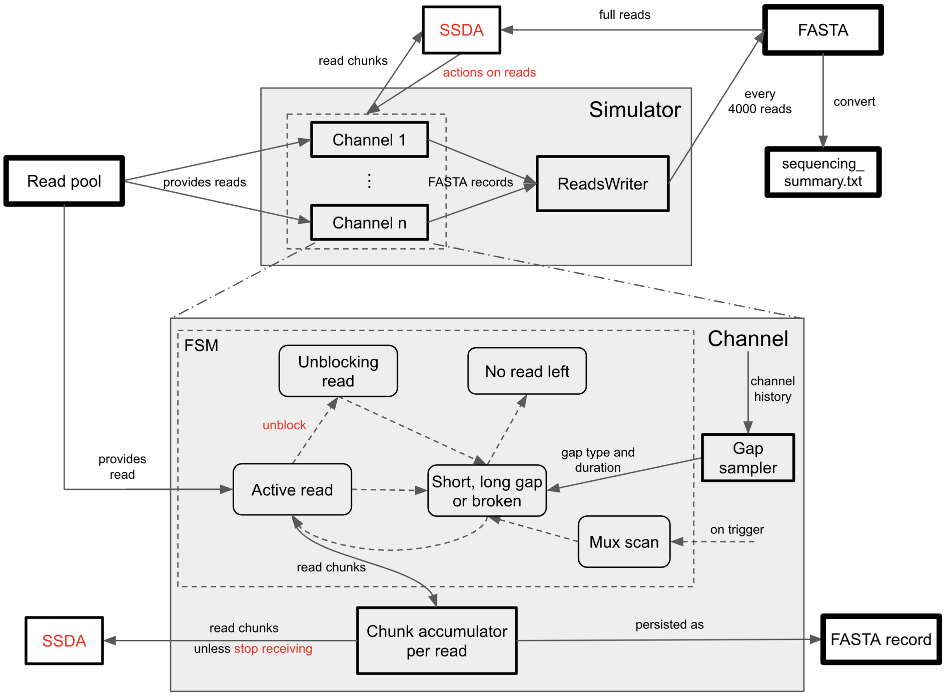

Fig. 2: The top shows *SimReadUntil* which consists of a set of channels, the bottom zooms into a single channel. Solid arrows mean data is flowing. Inputs and outputs are shown as square boxes with thick borders. **Top:** Simulator grouping a set of channels that fetch reads from a reads pool, e.g., a reads file generated with NanoSim and possibly shuffled. Finished reads are output as a FASTA file every 4000 reads. The FASTA output can be converted into a sequencing summary file (which is commonly output by ONT basecallers) since the FASTA header contains all relevant information. **Bottom:** Zoom into a single channel which is a finite-state machine (FSM) whose transitions (dashed arrows) between states (rounded boxes) happen either due to time or ReadUntil decisions (red) made by the selective sequencing decision algorithm (SSDA). The gaps are chosen from a gap sampler and can depend on the channel history with parameters tuned from an existing run. The mux scan can be triggered at any time (ejecting the element in-progress) and is followed by a gap once it finishes.

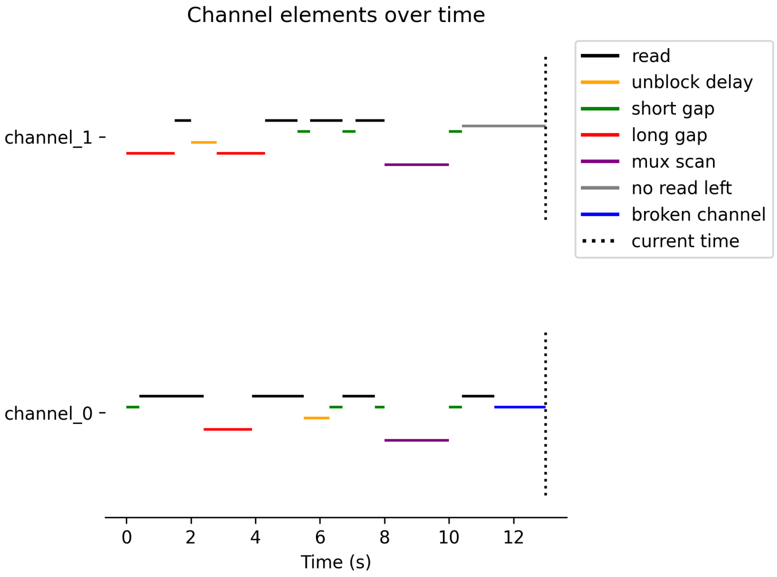

Fig. 3: Example of a simulation with two channels. Parameters were chosen to show all available channel elements. In real ONT runs, mux scans occur roughly every 90 minutes.

```
Examples:
chr-18_12681_aligned_proc3:68_F_5_13231_10
genome1-chr-6_236227_unaligned_proc5:16_R_0_16119_0
```

The read comes from the forward or reverse `strand` of `chromosome` in `genome` starting at 0-based position `ref_pos` with a length of `ref_len` on the forward strand of the reference, so it spans the half-open interval `[ref_pos:ref_pos+ref_len)` on the forward strand. Due to indels, the read length can be shorter or longer than `ref_len`. The prefix `{genome}-` (species) is only added in metagenome mode to distinguish chromosomes from different species. When a read is rejected (*unblock*), the `ref_pos` and `ref_len` are adapted proportionally. NanoSim adds flanking regions around the read consisting of random letters. When using a read delay (which stands for the adapter and poly A tail preceding the actual read), we remove the flanking regions, that is, we set `head_len` and `tail_len` to 0 unless otherwise stated. The read number `read_nb` is unique to each read and concatenates the process id and read number (as NanoSim uses multiprocessing)[2]. The read type is `aligned`, `unaligned` or `perfect` for reads with low error rate (as in the NanoSim training set), high error rate (90% such that alignment will likely fail), and without errors respectively.[3] Since NanoSim is rather slow, we recommend generating the reads (which can result in files on the order of 10GBs) and create the pysam `*.fai` fasta index files in advance.

Our simulator comes with post-processing tools that parse the read ids and plot the fraction of basepairs covered at least x times for various values of x over time per `[{genome}]-{chromosome}`. When the reference genome is large, we reduce the granularity during counting by taking blocks of basepairs for efficiency.

C. Parameter Extraction Methods

We set the parameters of the simulator based on the `sequencing_summary.txt` of an existing run. The sequencing summary is available from an ONT run after basecalling the reads from the raw signal. This provides information of the timings of the reads in each channel. We extract the following parameters:

- The number of basepairs per second `bp_per_second` is estimated as the median read speed.
- The read delay is the delay between the start of a read until sequencing the first nucleotide excluding the adapter sequence (which basecallers usually trim, see `https://timkahlke.github.io/LongRead_tutorials/FTR_P.html`), and can be computed as the difference between the template start and the read start. We explicitly model it because the returned chunk data is already basecalled. We set it to be the median over all observed delays.
- The unblocking delay is the duration during which the unblock voltage is applied to eject the read. This is typically set to a fixed value ≈ 0.1s (Kovaka et al., 2021).

We decided to set these parameters to constant values because they are mostly constant and to limit the number of parameters.

The gap sampler returns the length of the gap following a read as well as the gap type (long or short). Similarly to UNCALLED, we classify the gaps as short and long gaps. The threshold to determine this is the median plus five times the interquartile range of the gap lengths computed over all observed gaps across all channels.[4] A long gap continues after a mux scan, whereas a short gap does not. Gaps are the regions between reads in the interval 0 to the end time of the last read of a sequencing run. We have implemented the following gap samplers whose parameters are fitted from an existing run:

- `random_gaps` samples gaps from an arbitrarily chosen (fixed) distribution and does not extract parameters from an existing run. This is for debugging and prototyping new methods.
- `constant_gaps` computes the median of the short gaps and long gaps, respectively, and selects a long gap with a constant probability. Due to the skewed gap length distribution (towards long gap durations), setting the probability of a long gap to be equal to the fraction of long gaps is not appropriate because this means that the fraction of time spent in short gaps becomes larger. Rather, we require the fraction of time spent in long gaps to be the same as in the original run. For this to be the case, we set the probability of selecting a long gap to be $f_l = \frac{1}{1+\frac{t_l}{t_s}\left(\frac{1}{f}-1\right)}$, where t_s and t_l are the times of short and long gaps, respectively, and f is the fraction of long gaps among all gaps. We provide a derivation below. This approach is useful for rapid prototyping and for assessing SSDAs at peak throughput (when pores do not deteriorate) without biasing metrics too much towards a particular run. It is similar to Icarust which employs a constant (configurable) short gap defaulting to 0.4s. Unlike Icarust, we extract this delay from the data and distinguish short from long gaps.
- `gap_replication` replicates gaps similarly to UNCALLED. A channel consists of alternating active and inactive periods. The long gaps represent long inactive periods without any reads, the time between them corresponds to the active periods. The order and duration of active and inactive periods is replicated as in the real run. Within an active period, gaps between reads are sampled from the short gaps observed within that period. A period ends once its duration is exceeded. This waits until the read has finished, so an active period may be longer than in a real run. It is suited when we want to replicate an existing run as closely as possible, but still

[2] NanoSim originally returned the global read number by counting the number of reads generated so far across all processes. We modified it to instead return the process id concatenated with the read number, e.g., `proc3:68` means that it is the 68th read from process 3.

[3] For perfect reads, it is easier and more efficient to generate the reads directly in-memory as input to the simulator.

[4] This is different from UNCALLED which uses the median plus one standard deviation. Since the gap length distribution has many outliers, the standard deviation is not a very robust measure.

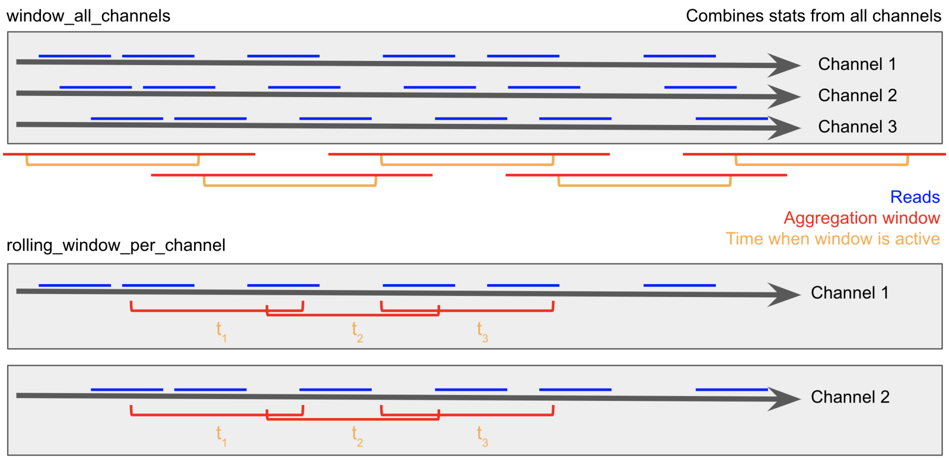

Fig. 4: Gap sampler methods extracting gaps between reads. Top: `window_all_channels` gap sampler with contiguous time windows that sample gaps from the aggregation windows containing them, so the aggregation windows between adjacent time windows overlap. The windows aggregate data over all channels, so well-performing and badly-performing channels are mixed. Bottom: `rolling_window_per_channel` gap sampler with rolling windows per channel (only showing 2 channels for simplicity). The per-channel sampling means less data is available, especially towards the end of the sequencing run.

allow for ReadUntil decisions to influence the run. This method is similar to UNCALLED and MinKNOW (which does not support unblocks properly). Since the `gap_replication` approach is exactly replicating an existing run, a time-dependent (blackbox machine learning based) SSDA may overfit to the existing run.

- `window_all_channels` defines contiguous time windows and samples the gaps from the corresponding time windows in the real run. To avoid abrupt changes, the gaps are sampled from overlapping windows, see Figure 4. The windowed approach ensures that gaps from the start and end of a sequencing run are not mixed because they have different statistics: gaps at the end are considerably longer because the pores deteriorate. The gaps are sampled from the data in the time window over all channels to have sufficient data, but this mixes good and bad channels.
- **Recommended method**: `rolling_window_per_channel` operates per channel and uses rolling windows compared to `window_all_channels`. It first samples the channel in the original run, then samples gaps in a rolling time window around the current timepoint in the corresponding channel, see Figure 4. It stops once the sequencing time of the channel is exceeded. The disadvantage of sampling gaps per channel is that there are fewer gaps in the window (as low as 1) especially towards the end when the channel is more likely to be inactive for long periods of time, possibly leading to poor statistics.

Channels deteriorate over time and some channels work better than others. To take this into account, all methods except `random_gaps` sample from the empirical distribution of sequencing time per channel. Once the time is exceeded (after a read has finished), the channel stops producing any new reads. The number of channels can be flexibly chosen except for `gap_replication` which returns "broken" channels that do not produce any reads once the original number of channels has been exceeded. The `constant_gaps` and `window_all_channels` methods sample from the discrete distribution of active channels only, i.e., channels having produced at least one read. The `rolling_window_per_channel` method is similar, except that it returns a broken channel with the same probability as in the existing run, for which it requires the full number of channels since the sequencing summary file only contains channels which have produced at least one read. We also experimented with fitting the time until a channel produces its last read to a distribution (beta, weibull_min), but this did not deliver equally good results in terms of basepairs and reads over the full run. Since loading the sequencing summary for parameter extraction takes some time (though <1 minute on a normal computer for a 200-500MB sized sequencing summary), we cache the simulation parameters.

We now derive the probability of sampling from a long gap to ensure that the fraction of time spent in long gaps corresponds to the fraction $0 \leq f \leq 1$ of an existing run. Assume we have sampled n gaps with $0 \leq x_l \leq n$ long gaps among them. Let t_s, t_l denote the expected length of short and long gaps respectively[5]. We want the following to hold in expectation:

$$f \cdot (x_l t_l + (n - x_l) t_s) = x_l t_l.$$

Note that we do not match the ratio, but multiply the denominator because the expectation of a ratio cannot be easily simplified with linearity (x_l is a random variable). Taking the expectation, we have $\mathbb{E}[x_l] = f_l n$ given that we independently sample a long gap with

[5] Here, we derive it for the general case when the gap length is not necessarily constant, e.g., sampled from some distribution. In the setting above, we extract the medians, so the short and long gap times are constant.

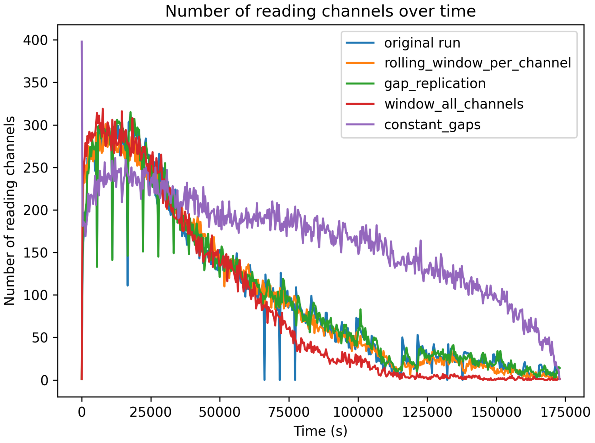

Fig. 5: Actively reading channels over time. The original sequencing run is `original_run` .

probability f_l. Inserting this, we solve for f_l:

$$\frac{1}{f_l} = 1 + \frac{t_l}{t_s}\left(\frac{1}{f} - 1\right).$$

As a sanity check, the case $t_s = t_l$ corresponds to $f_l = f$, $t_l/t_s \to \infty$ to $f_l \to 0$, $t_l/t_s \to 0$ to $f_l \to 1$, $f \to 0$ to $f_l \to 0$, $f \to 1$ to $f_l \to 1$.

C.1. Comparison of Parameter Extraction Methods from a Real Run

In this section, we extract parameters from a real run and compare the statistics of the simulated run to the original run, see `replicate_run.py`. For this, we take the sequencing summary file provided by UNCALLED (Kovaka et al., 2021) mentioned in the README of their GitHub: `https://labshare.cshl.edu/shares/schatzlab/www-data/UNCALLED/simulator_files/20190809_zymo_seqsum.txt.gz` from a sequencing run of the Zymo mock community. We run the simulated run without selective sequencing since selective sequencing affects the statistics like read duration. Since mux scans potentially introduce long gaps that we do not want to model, we first find and remove mux scans with a script that we have adapted from UNCALLED.[6] We then extract the read duration from the sequencing summary file and generate reads such that their lengths match the extracted read durations (at 450 bp s^{-1} speed). The simulator forwards each channel in parallel. The implementation is parallelized with *joblib* and faster the more cores are available. Note that we only parallelized the code when ReadUntil functionality is not used, which is the case here.

We compare the different parameter extraction methods. More figures are available in the supplementary material. Figure 5 compares the number of actively reading channels at regular timepoints. We see that the `gap_replication` closely matches the original run `original_run` . The `rolling_window_per_channel` also works very well while offering some deviation from the existing run. The `window_all_channels` works well at the beginning, less at the end. A reason may be that the method averages across all channels, thus combining information from channels with different channel healths. The `constant_gaps` strategy does not perform well, especially at the end. Figure 6 compares the number of reads and sequenced basepairs over time. We see that the qualitative behavior is similar to before. The constant gaps strategy works well at the beginning, but less so at the end because it does not take into account that read gaps get longer over time due to pore deterioration. The other methods have similar behavior as the original run, while introducing some randomness by sampling the gaps (which is not the case for `gap_replication`). Figure 7 compares the channel statistics for the various methods. As expected, the `original_run` and `gap_replication` have (almost) identical histograms, the `rolling_window_per_channel` method also has similar statistics. All methods except `constant_gaps` have very similar means and medians. The statistics of the `constant_gaps` method differ substantially, substantiating the claim about it being a good starting point, but not faithful replication of an existing run. The `window_all_channels` method shows some differences for the time spent sequencing, which may come from it mixing gaps from channels with different pore healths. If a channel breaks after a long gap, its sequencing end time ends at the start of the long gap, which can lead to channels terminating much earlier as seen for the `window_all_channels` method.

D. Use Case: SimReadUntil with ReadFish

We present an example on how to use our simulator with ReadFish (Payne et al., 2021), see `enrich_usecase.py`. ReadFish takes selective sequencing decisions by first basecalling raw reads and then mapping the basecalled reads to a reference with *minimap2* (Li, 2018). Similar to Figure 1 of the ReadFish paper (Payne et al., 2021), we take the human reference genome and enrich chromosomes 1-8, 9-14 and 16-20 respectively, referred to as experimental conditions. The control condition performs normal sequencing (with no selective sequencing decisions). We extract simulation parameters from the same Zymo mock sequencing summary file as before (from UNCALLED (Kovaka

[6] This script is not perfect, which explains jumps in the plots of actively reading channels around the mux scan intervals. The mux scans occur roughly every 90 minutes, but not exactly.

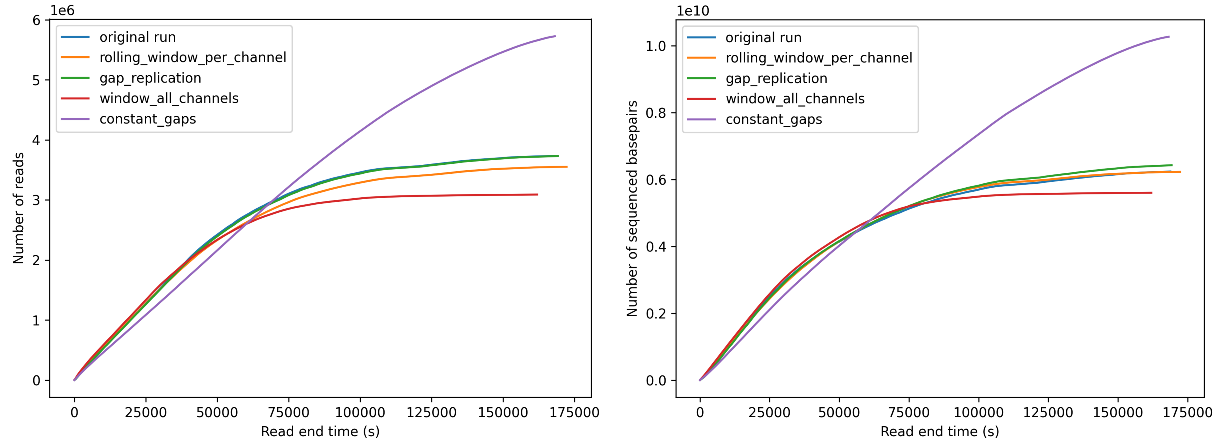

Fig. 6: Total number of reads and total number of sequenced basepairs across all channels over time. The original sequencing run **original_run** and the replication run **gap_replication** overlap as expected.

et al., 2021)) using the `rolling_window_per_channel` parameter extraction method after removing mux scans. We simulate 512 channels at different acceleration factors for a simulation time of 30h with 512/4 channels per condition. Since read rejections discard the rest of a read, full reads are not available from the ReadFish experiment. Therefore, we generate reads using a modified version of NanoSim with their error model pretrained on human reads, as explained in Section B.1. We use no flanking around the actual reads, but instead add a read start delay. We did not notice a significant difference when including flanking regions which indicates that the minimap2 aligner can handle this well. We modified NanoSim to extract reference fragments from a uniform distribution in the range $[12000, 16000]$ (to be similar to ReadFish's reported median length of 15000 bps) since its `median_len` CLI parameter did not work as promised. The actual read can be shorter or longer due to indels. All reads with lengths outside the range of $[400, 20000]$ were discarded. We use an alignment rate of 2 which means that the ratio of alignable to unalignable reads is 2:1. The minimap2 aligner manages to align 81% of the alignable reads to the correct chromosome. Since NanoSim is rather slow, we generated reads in advance using different seeds in parallel.

In each iteration, ReadFish requests new read chunks from all 512 channels (batch size 512), basecalls them, maps them to the reference with `minimap2`, makes decisions about each of them, communicates the decisions back in one batch via the ReadUntil API and waits a minimum time between iterations (throttle) to avoid overloading the sequencer/simulator. ReadFish needs to be adapted because it connects to the sequencer via ONT's ReadUntil Python client and receives raw read data whereas our simulator returns basecalled data. We modified ReadFish starting from the original commit `ab3b25a7ad7aace69979436cf679c054d82585d5`. We implement a fake basecaller that returns the basecalled data as-is since our modified ReadUntil API returns basecalled data rather than raw signal data. Since basecalling constitutes a fundamental bottleneck of ReadFish, we introduce a time delay per basecalled basepair that we estimated from benchmarking experiments (`https://hackmd.io/@Miles/HJUnkIeOK`). A GPU can call roughly 2.6×10^7 samples s^{-1}, where a sample refers to the ONT raw signal at 4 kHz producing roughly 450 bp s^{-1}. This means $(4\,\mathrm{kHz}/450\,\mathrm{bp\,s}^{-1})/(2.6 \times 10^7\ \mathrm{samples\,s}^{-1}) = 3.4 \times 10^{-7}\,\mathrm{s\,bp}^{-1}$. This can be adapted to measure how ReadFish would perform when basecalling with CPUs only. In accelerated mode, we divide this number by the acceleration factor.[7] Moreover, we observed that the throttle must be adapted in accelerated mode because sequencing decisions otherwise get communicated too late. We keep the batch size the same and divide the default throttle of 0.1 s by the acceleration factor. Depending on the genome size, we observed that mapping is too slow at acceleration factors greater than 2-3. Since NanoSim gives access to the ground-truth mapping of the reads encoded in the read ids (see Section B.1), we can skip `minimap2` alignment and replace it by a fast read id parser. This allows to run at greater acceleration factors.

We first check that the latency of the simulator is low enough by running `readfish unblock-all` which rejects reads as soon as it receives chunks from a new read. This can be useful for deciding on the acceleration factor at which to run the simulator. At acceleration factor 1, most reads are rejected after 250-300 bps. This increases up to 450 bps at acceleration factor 10.

We now proceed to the enrichment experiment using `readfish targets`. We obtain a separate set of plots for each condition. For exemplary purposes, Figure 8 provides plots for the condition enriching chromosomes 9-14, using an acceleration factor of 2 and `minimap2` mapping. The occasional little kinks in the curves happen due to computer issues related to running in a shared cluster. Most reads from the enrichment target (chr9-14) are set to stop receiving and fully sequenced, whereas most off-target reads are rejected early on. Deviations happen due to alignment errors or decisions arriving too late. We see that most reads get rejected after roughly 500 bps. This is similar to rejection lengths commonly observed in real setups. Unaligned reads get rejected after 12 chunks, which is roughly $(12 + 1) \cdot 200 = 2600$bp, adding +1 to communicate the read decision back. Figure 9 shows the fraction of the on- and off-target chromosomes covered at least x times, which demonstrates that enrichment is acquiring more data from the target regions. The fraction

[7] Adapting the basecall delay makes sense if the goal is to speed up experiments which should also speed up downstream tools like ReadFish (and ideally speed up alignment as well, e.g., by using a ground-truth aligner). If the goal is to check if ReadFish can handle higher sequencing speed, the basecall delay should not be modified.

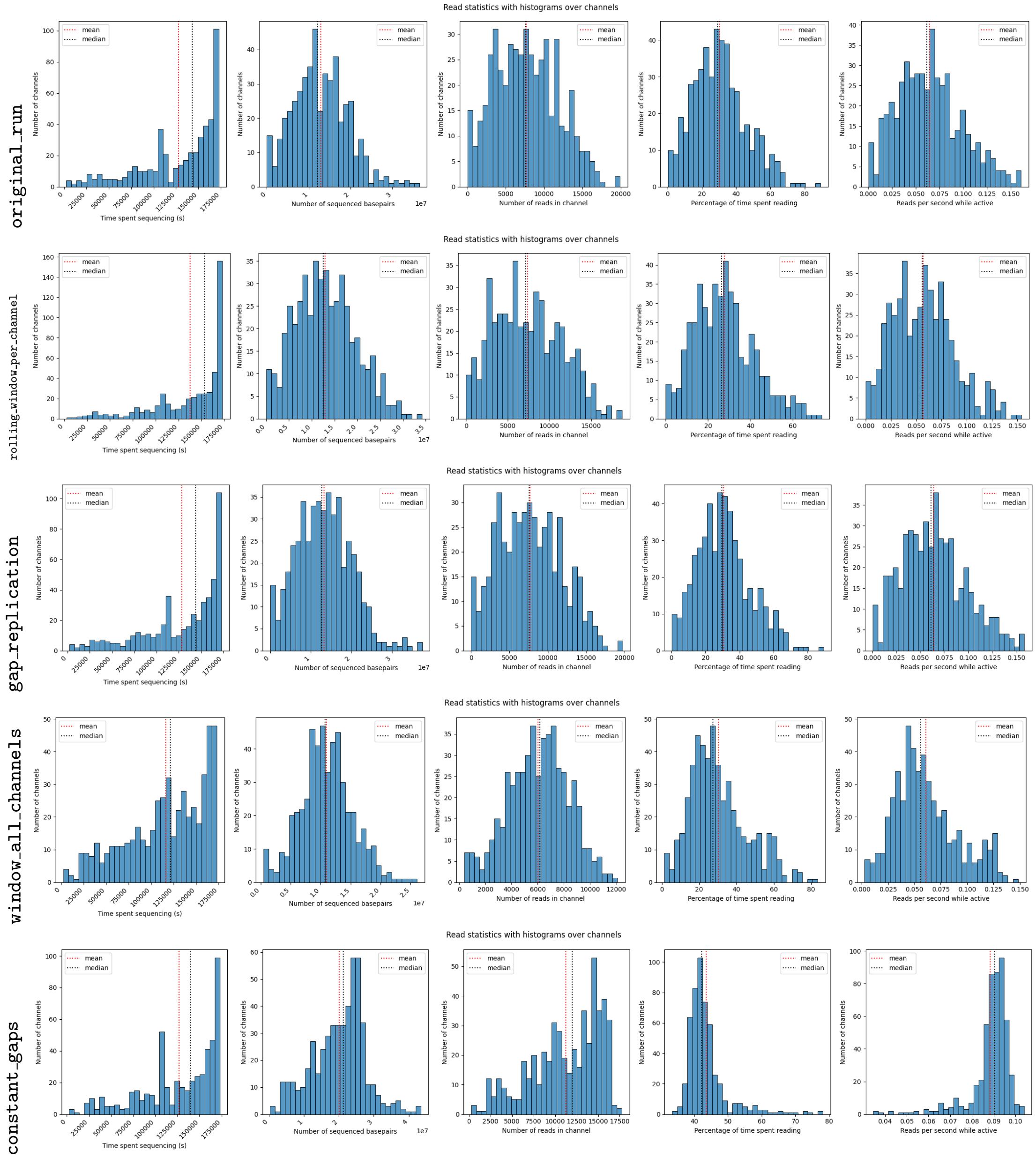

Fig. 7: Statistics across channels for the **`original_run`** (original run), **`rolling_window_per_channel`** (recommended method), **`gap_replication`** (replication), **`window_all_channels`** (mixing all channels) and **`constant_gaps`** (constant gaps) parameter extraction methods (top to bottom). The percentage of time spent reading only accounts for the percentage until the channel produces its last read.

covered is computed by dividing each chromosome into blocks of size **`blocksize`** (except for the last which is shorter). Whenever a block intersects with a read, we increment that block's count by the number of basepairs intersecting with the read. After adding all reads up to time t, we can compute the fraction of blocks with average coverage at least 1, 2, 3, 4 which is shown in the figure comparing the target to the non-target chromosomes. We use a **`blocksize`** of 1000 which greatly speeds up computations with respect to **`blocksize`** 1.

Table 2 compares all conditions and computes relative enrichment (RE) and absolute enrichment (AE). The caption includes a definition of these quantities (Martin et al., 2022). RE measures how selective sequencing changes the sample composition and can be used to check that enrichment is working. AE compares the yields obtained in a control run with those obtained in a selective sequencing run. It is most relevant to assess how much selective sequencing helps with getting the desired data as fast as possible. Rejections add an extra delay where no data is acquired and are detrimental to pore health and decrease overall flow cell yield, so that the AE is usually significantly lower than relative enrichment. In practice, different channels perform differently, so it is recommended to compute the AE over a set of channels. In Table 2, we see that RE is (roughly) proportional to the maximum achievable relative enrichment (1/fraction of target). The mismatch happens both due to having to sequence the initial fragments of all reads to come up with a decision, and

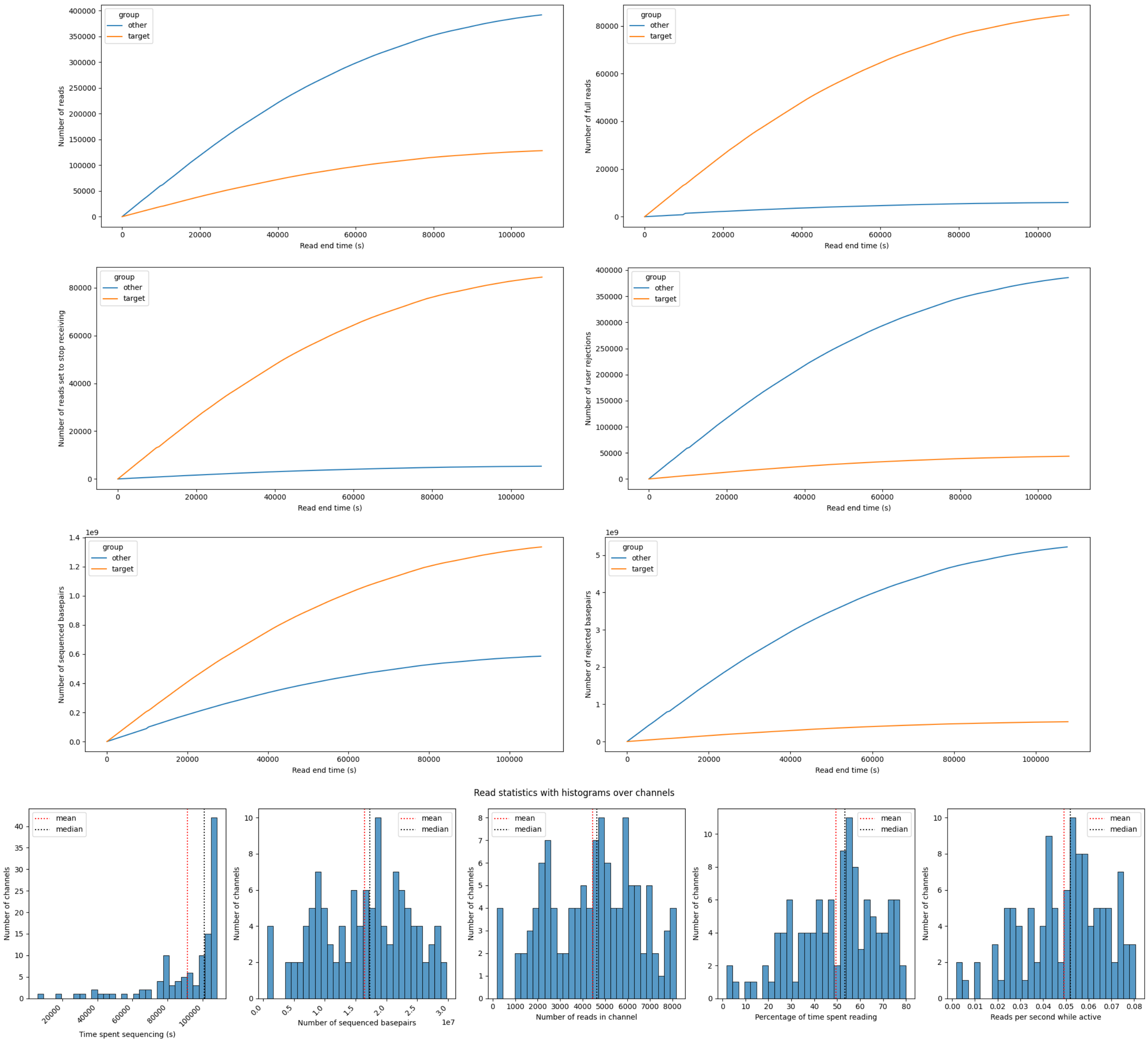

Fig. 8: Combining the simulator with ReadFish to enrich for chromosomes chr9-14 (target vs other). The data is randomly subsampled. Number of reads, full reads (reads that were not rejected), reads set to *stop_receiving*, rejected reads, number of sequenced and rejected basepairs, read stats by channel. Continued in next figure.

incorrect decisions due to mapping errors. Slightly more precise estimates of the maximum achievable RE can be obtained by using the Shiny app (`https://sr-martin.shinyapps.io/model_app/`) from (Martin et al., 2022) which assumes perfect basecalling and mapping and takes into account the time spent sequencing the initial fragments of reads as well as constant read gaps, but not the reduction of pore occupancy due to rejections. We ran the Shiny app with the following parameters: average read length 14000 bps, species abundance 49.6%, 24.7%, 12.5%, sequencing speed 370bp/s, decision time 1s, capture time 0.5s. The deviations could be explained by only 66% of all reads aligning (ratio 2:1). AE is most affected by the reduction in yield. We observe that AE increases only by 50% rather than the ideal 100%, when the target region decreases by a factor of 2. In addition to the mentioned factors which decrease RE, this can be explained by the reduced flowcell yield compared to the control run caused by the extra read gaps and unblock delays. We also observe that the predicted AEs are quite different from the AEs reported in the ReadFish paper (Payne et al., 2021). Reasons for this are different simulation parameters (read gaps, read speeds), not modeling pore degradation due to rejections, different alignment rate of reads due to NanoSim read simulation. It is out of scope of this work to model pore degradation, which is not well understood yet (Payne et al., 2021; Martin et al., 2022). Each condition is characterized by a different rate of rejections. We recommend fitting simulation parameters to each condition separately. We requested the sequencing summary file for the ReadFish experiment from the authors, but did not receive

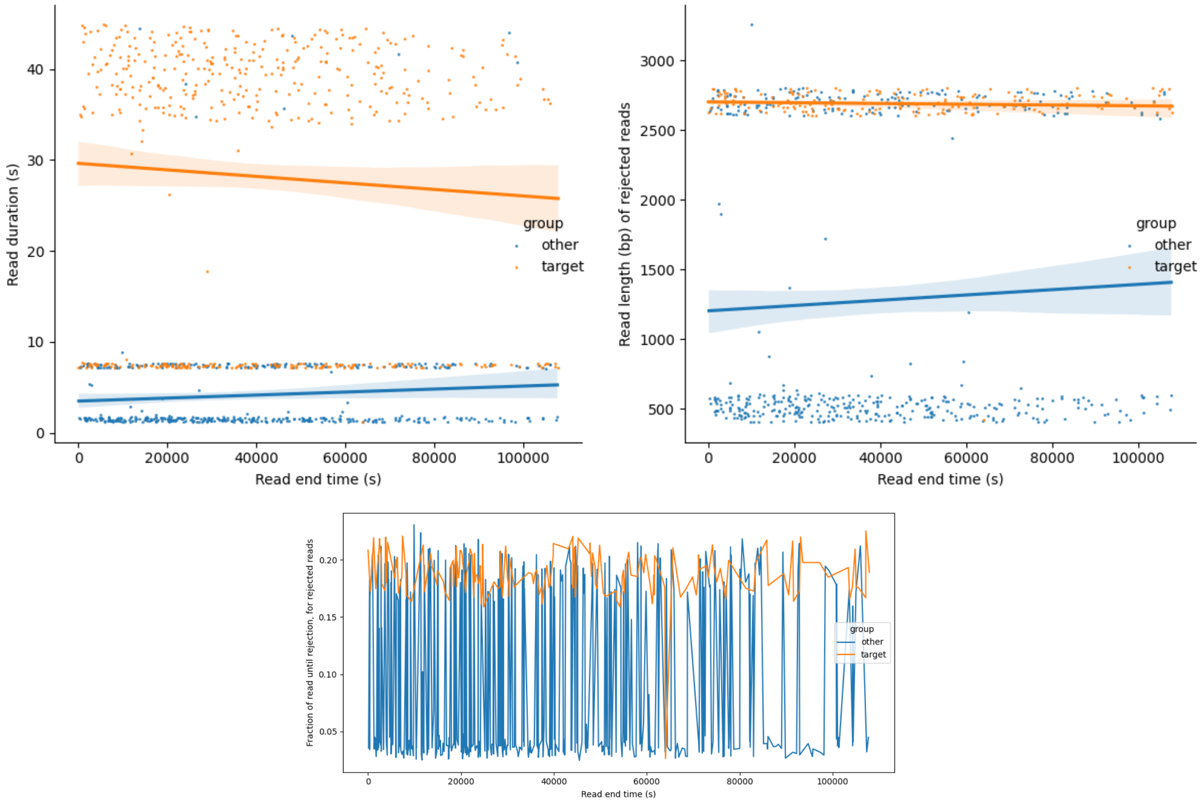

Fig. 8: (Continued) Duration of reads, length of rejected reads, fraction of read until it got rejected for rejected reads.

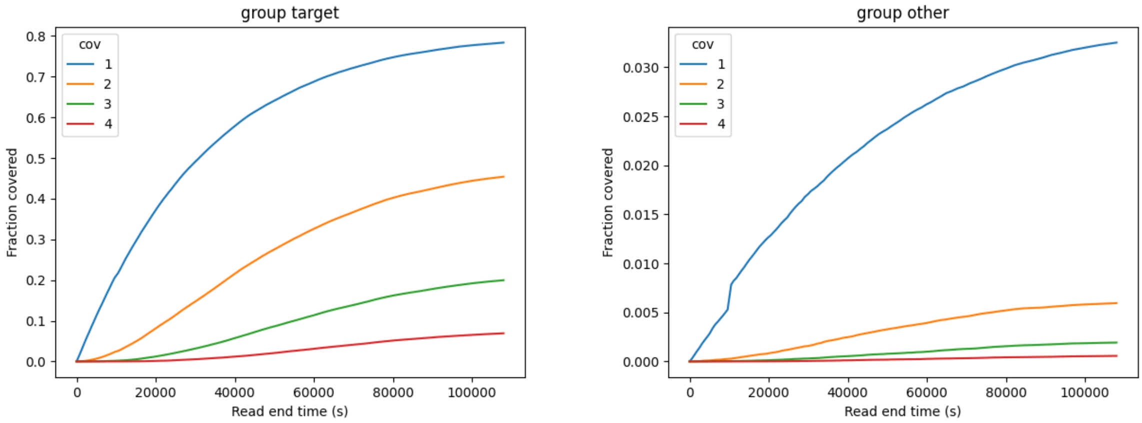

Fig. 9: Combining the simulator with ReadFish to enrich chromosomes 9-14 (target vs other): fraction of basepairs covered at least x times for $x = 1, 2, 3, 4$ as the sequencing run progresses. The coverage of the target is significantly higher than the coverage of the off-target. Note that the number of basepairs of chr9-14 is roughly 1/3 of the number of basepairs of the off-target, but this does not affect the conclusion. The kink happens because the experiment was run on a shared cluster.

any response despite an initial response. Our simulation parameters may differ from the ReadFish run. For example, we extracted a read speed of $370\,\mathrm{bp\,s^{-1}}$ whereas ReadFish may be closer to $450\,\mathrm{bp\,s^{-1}}$. It is also slightly surprising that the AE does not change at all between the chr9-14 and chr16-20 conditions, which may point to some experimental difficulties that are challenging and questionable to model. Moreover, read alignment rates can be as low as 10% which is much lower than our simulated rate of 66%. This could be the case for the ReadFish run.

The existing raw-signal simulator UNCALLED (Kovaka et al., 2021) reports accurate prediction of the AE achieved in a new run when they fit the parameters from an existing run following a similar experimental protocol for both runs. We fitted the simulation parameters from the UNCALLED run, whereas the ReadFish run comes from a different lab, so the sequencing performance can be quite different, especially at the time the (Payne et al., 2021) experiments were conducted. It is well known that flow cell variability differs considerably (Martin et al., 2022; Payne et al., 2021), so this comparison has to be taken with a grain of salt rather than as an argument against our simulator.

Condition	ReadFish		Simulator			
	Total Yield	AE	Total Yield	On-Target Yield	RE (max)	AE
Chr 1-8 (49.6%)	2.79Gb (1.24x)	1.5	2.15Gb (1.24x)	1.86Gb	1.75 (2.02, 1.94)	1.41
Chr 9-14 (24.7%)	1.84Gb (1.89x)	2	1.92Gb (1.39x)	1.34Gb	2.81 (4.05, 3.63)	2.03
Chr 16-20 (12.5%)	1.22Gb (2.84x)	2	1.74Gb (1.54x)	0.891Gb	4.11 (8, 6.24)	2.67

Table 2. Comparison of the simulated run with the original ReadFish run from Figure 1 in (Payne et al., 2021). Absolute enrichment (AE), relative enrichment (RE). The total yield column also indicates in parentheses the reduction in yield with respect to the control condition. The original ReadFish run has a total yield of 3.47Gb for control. The simulated control condition has total yield of 2.67Gb, 1.32Gb come from chr1-8, 0.657Gb from chr9-14 and 0.334Gb from chr16-20. Off- and on-target yield are the number of sequenced on- and off-target basepairs respectively. The relative enrichment (Martin et al., 2022) is computed as the ratio of (on-target yield/total yield)/(l_x/l_{genome}) with l being the length and x = chr1-8 for example. For RE, the maximum possible RE is indicated in parentheses, with the simple model 1/target_fraction (e.g., $1/0.496 = 2.02$) and the slightly more complex Shiny model from (Martin et al., 2022)). Absolute enrichment is the ratio of (on-target yield in condition)/(target yield in control). For ReadFish, the absolute enrichment was extracted from their Figure 1c. $512/4 = 128$ channels were assigned to each condition.

Experiment								
with unaligned	mapper	acceleration	RE (chr1-8)	AE (chr1-8)	RE (chr9-14)	AE (chr9-14)	RE (chr16-20)	AE (chr16-20)
no	fake	10	1.94	1.63	3.65	2.66	6.39	4.0
no	minimap2	5	1.89	1.59	3.42	2.56	5.90	3.78
yes	minimap2	2	1.75	1.41	2.81	2.03	4.11	2.67
yes	minimap2	3	1.75	1.40	2.82	2.07	4.13	2.72
yes	minimap2	5	1.75	1.40	2.82	2.04	4.14	2.70
yes	minimap2	10	1.22	1.06	1.49	1.29	1.73	1.48

Table 3. Absolute enrichment (AE) and relative enrichment (RE) when changing simulator settings for the enrichment experiment with 4 conditions: including unaligned reads, using the minimap2 mapper vs (ground-truth) fake mapper, different accelerations. Unaligned reads are generated with NanoSim with an alignment rate of 2, i.e. NanoSim generates a ratio of 2:1 of aligned vs unaligned reads. All experiments ran for a simulated time of 30h. When using aligned reads only (second row), the predicted REs are closer to those maximum achievable REs given in Table 2 because the modelling assumptions are better met.

We want to point out that for fixed simulated parameters, the simulator can be effectively used to compare selective sequencing algorithms, where the difference of the AE matters rather than the absolute values. It is to future experimental work to show that the sim2real gap is acceptable for identifying good algorithms. Table 3 computes AE and RE for different simulator settings. We see that ReadFish can handle acceleration factors 2, 3, 5 quite well, but AE and RE drop significantly at acceleration factor 10. Moreover, running with only aligned reads or using ground-truth alignment increases the AE and RE, as expected. Similar experiments could be conducted to assess the ReadFish performance drop when using CPUs instead of GPUs for basecalling.

References

C. Firtina, N. M. Ghiasi, J. Lindegger, G. Singh, M. B. Cavlak, H. Mao, and O. Mutlu. Rawhash: Enabling fast and accurate real-time analysis of raw nanopore signals for large genomes. *bioRxiv*, pages 2023–01, 2023.

S. Kovaka, Y. Fan, B. Ni, W. Timp, and M. C. Schatz. Targeted nanopore sequencing by real-time mapping of raw electrical signal with uncalled. *Nature biotechnology*, 39(4):431–441, 2021.

H. Li. Minimap2: pairwise alignment for nucleotide sequences. *Bioinformatics*, 34(18):3094–3100, 2018.

Y. Li, R. Han, C. Bi, M. Li, S. Wang, and X. Gao. Deepsimulator: a deep simulator for nanopore sequencing. *Bioinformatics*, 34(17): 2899–2908, 2018.

M. Loose, S. Malla, and M. Stout. Real-time selective sequencing using nanopore technology. *Nature methods*, 13(9):751–754, 2016.

S. Martin, D. Heavens, Y. Lan, S. Horsfield, M. D. Clark, and R. M. Leggett. Nanopore adaptive sampling: a tool for enrichment of low abundance species in metagenomic samples. *Genome biology*, 23(1):11, 2022.

R. J. Munro, A. Payne, and M. W. Loose. Icarust, a real-time simulator for oxford nanopore adaptive sampling. *bioRxiv*, pages 2023–05, 2023.

A. Payne, N. Holmes, T. Clarke, R. Munro, B. J. Debebe, and M. Loose. Readfish enables targeted nanopore sequencing of gigabase-sized genomes. *Nature biotechnology*, 39(4):442–450, 2021.

J. Quick. ncov-2019 sequencing protocol v3 (locost) v. 3. *Spanish J Leg Med*, pages 1–17, 2020.

A. Sneddon, A. Ravindran, N. Hein, N. E. Shirokikh, and E. Eyras. Real-time biochemical-free targeted sequencing of rna species with riser. *bioRxiv*, pages 2022–11, 2022.

J.-U. Ulrich, A. Lutfi, K. Rutzen, and B. Y. Renard. Readbouncer: precise and scalable adaptive sampling for nanopore sequencing. *Bioinformatics*, 38(Supplement_1):i153–i160, 2022.

Y. Wang, Y. Zhao, A. Bollas, Y. Wang, and K. F. Au. Nanopore sequencing technology, bioinformatics and applications. *Nature biotechnology*, 39(11):1348–1365, 2021.

C. Yang, J. Chu, R. L. Warren, and I. Birol. Nanosim: nanopore sequence read simulator based on statistical characterization. *GigaScience*, 6(4):gix010, 2017.
